# A dose response analysis of a specific bone marrow concentrate treatment protocol for knee osteoarthritis

**DOI:** 10.1186/s12891-015-0714-z

**Published:** 2015-09-18

**Authors:** Christopher J. Centeno, Hasan Al-Sayegh, Jamil Bashir, Shaun Goodyear, Michael D. Freeman

**Affiliations:** 1 Centeno-Schultz Clinic, 403 Summit Blvd Suite 201, Broomfield, CO 80021 USA; 2 Forensic Research and Analysis, Portland, OR USA; 3 Oregon Health & Science University, Portland, OR USA

## Abstract

**Background:**

Prior studies describing the treatment of symptomatic knee osteoarthritis with injections of bone marrow concentrate have provided encouraging results. The relationship between the cellular dose contained within the bone marrow concentrate and efficacy of the treatment, however, is unclear. In the present study we describe clinical outcomes for symptomatic knee osteoarthritis in relation to higher and lower cell concentrations contained within a bone marrow concentrate treatment protocol.

**Methods:**

Data from an ongoing patient registry was culled to identify 373 patients that received bone marrow concentrate injections for the treatment of 424 osteoarthritic knee joints. The clinical scales for these patients were assessed at baseline and then tracked post-procedure at 1, 3, 6 and 12 months, and annually thereafter. Tracked outcomes included the numeric pain scale; a lower extremity functional questionnaire; an International Knee Documentation Committee scale; and a subjective improvement rating scale. Using pain and functional outcome measures, a receiver operating characteristic analysis was used to define an optimal clinical outcome threshold at which bone marrow nucleated cell count could be divided into either a lower or higher cell count group within a treatment protocol.

**Results:**

The lower and higher cell count groups were defined using a threshold of 4 × 10^8^ cells. There were 224 and 185 knee joints treated in the lower (≤4 × 10^8^) and higher (>4 × 10^8^) cell count groups respectively. Most joints were diagnosed with early stage knee osteoarthritis. Both the lower and higher cell count groups demonstrated significant positive results with the treatment for all of the pain and functional metrics. The higher cell count group reported lower post treatment numeric pain scale values, in comparison with the lower cell count group (1.6 vs. 3.2; *P* < 0.001). No significant differences were detected for the other metrics, however.

**Conclusions:**

Improved function and reduced pain was observed in patients treated with a bone marrow concentrate protocol regardless of cellular dose; however, patients receiving a higher concentration of cells reported a better pain outcome in comparison with the lower dose group. These preliminary findings suggest that cell dose may be an important factor governing clinical outcomes in autologous bone marrow concentrate treatment of knee osteoarthritis. Further studies using a larger patient population may help elucidate these findings.

## Background

Osteoarthritis of the knee is a common and progressive joint disorder characterized by gradual deterioration of the articular cartilage and inflammation of the adjacent tissues. An estimated 9 million people in the United States suffer from symptomatic knee osteoarthritis, and depending on disease severity, management ranges from conservative treatment to surgical intervention, including total knee replacement (TKR) [1]. In recent years the number and cost of TKR has increased dramatically; from 305,000 procedures costing an average of $25,500 per surgery in 2001 to 610,000 procedures costing an average of $52,000 per surgery in 2012 [2]. TKR is associated with significant complications, including deep vein thrombosis and infection, as well as post-surgical joint stiffness and muscle atrophy [3, 4]. Other lower risk surgical procedures are available as stopgap measures prior to TKR, such as arthroscopic meniscectomy, but such procedures are demonstrably lacking in long-term efficacy when compared with sham control procedures [5].

Non-surgical approaches to treating knee osteoarthritis, such as percutaneous injection of biological substances (e.g. hyaluronic acid and platelet-rich plasma (PRP), and Bone marrow derived nucleated cells (BMNCs)), represent a lower cost and lower risk alternative to surgery [6]. Clinical studies of intra-articular injection of PRP or BMNCs into the knee have demonstrated reduction of pain and improved function [7, 8].

In humans, nucleated cells are isolated from the aspirate of bone marrow that is typically harvested from the superior iliac crest of the pelvis via trocar [9]. The composition of these nucleated cells is diverse including Mesenchymal Stem Cells (MSCs), Hematopoetic Stem Cells (HSCs), Monocyte Precursor cells, Macrophages, T cells, B cells, Dendritic Antigen Presenting Cells, Natural Killer Cells and Neutrophils [10–12]. The action of these cells, acting both in isolation and symbiotically, once introduced into arthritic joints may help improve pain and function by replenishing damaged joint structures and providing a mediation of catabolic immune response, thus alleviating the symptoms and progression of the disease [12, 13].

MSCs are multipotent with the capacity to differentiate into various cell types from a mesodermal origin including cartilage, ligament, tendon, bone and fat [13]. Prior animal studies have demonstrated cartilage healing after joint osteoarthritis treatment with high dose expanded MSCs [14–16]; however, Bone Marrow Concentrate (BMC) contains only a fraction of the MSCs described in these studies [17]. Interestingly, other studies have demonstrated comparable results with direct application of BMNCs without the isolation and expansion of MSCs [10]. These observations raise numerous questions including to what extent the other nucleated cells in the bone marrow affect tissue healing and whether there is an optimal BMC cell dose for the most efficacious treatment of joint osteoarthritis.

BMC protocols have been employed throughout the growing field of Regenerative Medicine in the treatment of osteoarthritis [8, 13]. These protocols often contain PRP or Platelet lysate (PL), which enhance both bone marrow and adipocyte derived MSCs proliferation when used as a culture medium in vitro [18, 19]. Laden with growth factors [13], which have been show to influence MSC differentiation toward chondrocytes [19, 20], PRP and PL increase the activity of MSCs.

There is significant variability in MSC content of the bone marrow from person to person [21], hence providing the correct autologous dose to a joint could be important. Traditionally colony forming units have been used as a proxy for MSC dose [22, 23], but this technique can’t be used in real time to adjust the amounts injected at the bedside as it requires several days of culture for colony formation. Alternatively, flow cytometry could be used, but this technology is expensive and takes a dedicated lab staff to run, and is thus impractical for office based, clinical use [24]. Nucleated cell count may be a solution for office based use as the count is easy to obtain at the bedside and several studies show that it is a reasonable proxy for MSC dose [25, 26]. In addition, having nucleated cell count data readily available before cells are re-injected into a joint could allow for adjustments of the dose.

In the present observational study using registry data, we examine the effectiveness of using a specific lower and higher BMNC dose injection protocol for the treatment of knee osteoarthritis. We also analyze the clinical outcome differences between the two doses.

## Methods

### Setting and participants

Outcomes data for this study was derived from a previously reported treatment registry of patients receiving autologous MSCs, BMC, or PRP for the treatment of joint disorders including among others, knee, hip, and shoulder joints [25, 27–30]. The registry protocol was approved by a Institutional Review Board (HHS OHRP #IRB00002637). All subjects (or their guardians if they were under 18 years old) were required to undergo an informed consent process and sign an informed consent form before they enter the registry. In the current study, patients presenting to one clinic with symptomatic knee osteoarthritis, as determined by Magnetic Resonance Imaging (MRI), and knee pain complaints with decreased function were included for analysis. Knee osteoarthritis on MRI was defined as any abnormalities of the cartilage, bone, or meniscus for example: chondral or osteochondral defects, chondral loss, meniscus tears, bone marrow lesions, joint space narrowing, bone marrow lesions, or bone spurring. All MRI images were read, and severity grades were determined by treating physicians. All patients who were treated with BMC injections, combined with PRP and PL, were included in the study.

Patients enrolled in the treatment registry were prospectively followed using an electronic system, ClinCapture software (Clinovo Clinical Data Solutions, Sunnyvale, California) that generates an automated, post-treatment, questionnaire for evaluation at 1, 3, 6 and 12 months, and annually thereafter. Registry staff compiled all data, as well as contacted patients that failed to respond to the electronic survey.

### Procedure description

A detailed description of BMC aspiration and injection procedures has been previously published [31]. Briefly, two weeks prior to undergoing BMC injection, patients were restricted from use of corticosteroids and non-steroidal anti-inflammatory drugs (NSAIDs) as this reduces healing. On the day of the procedure, approximately 10–15 cc of whole bone marrow aspirate was harvested from 6 to 8 bone sites (approximately 3–4 on each side) of the patients’ Posterior Superior Iliac Crest. Under sterile conditions, the whole bone marrow aspirate was sequentially centrifuged, and resultant nucleated cells contained within were isolated for injection. Concomitantly, patient-derived heparinized venous blood was used to isolate PRP, and prepare PL by freeze-thawing the PRP. Using ultrasound or fluoroscopy to guide needle placement, a nucleated cell preparation of varying dose, contained within varying volume, and standardized volumes of PRP and PL were delivered to both the intra-articular space as well as painful or damaged structures of the knee. For example, if a meniscus tear was detected upon MRI or ultrasound examination, then in addition to the intra-articular space, the meniscus tear area was also injected with the nucleated cell preparation.

Post-operatively, patients with one compartment dominant disease (i.e. a single site within the knee joint) were provided with activity instructions and fitted with an off-loader brace that was to be worn for duration of 6 weeks for all weight bearing activity. Patients with patella-femoral compartment disease were fitted with a patella brace and instructed to avoid bearing full weight on the treated extremity for several days, then resume full weight bearing as soon as feasible. Full activity was gradually implemented over a 6-week period. Although patients were encouraged to undergo physical therapy, it was not required nor controlled.

### Nucleated cell dose

The predictor of interest in this study was the total nucleated cell count contained in the bone marrow aspirate. The number of nucleated cells in each patient sample was manually counted under a microscope (National Optical, Schertz, TX) using a hemocytometer (Reichert Bright-Line, Hausser Scientific, Horsham, PA). Prior to counting, red blood cells were lysed by diluting 5 μL of sample in 995 μL of sterile, distilled water (Life Technologies, Grand Island, NY). Each sample was counted four times and the average calculated. The total nucleated cell count for injection was determined by multiplying the dilution factor, the volume of the hemocytometer, and the final volume of the sample.

### Covariates

An examination of potentially confounding variables included: age, gender, BMI, severity of disease, and follow-up time in months. The baseline severity of osteoarthritis, as well as candidacy for the procedure, was graded by the treating physician using the Kellgren-Lawrence (KL) scale, where KL1 represented a “Good” candidacy grade, KL2 represented a “Fair” grade, and KL3 and 4 signified a “Poor” grade [32]. These categories were based on the imaging-determined disease severity [33]. This included the use of MRIs and AP standing radiography. BMI and follow-up time in months were examined as continuous numeric variables.

### Outcomes of interest

In this study, four clinical scales were used to measure outcomes of pain or functional improvement. The follow-up duration for each scale was measured as a score reported by the patient beginning from the date of the procedure (i.e. BMC injection) to the date of the last follow-up.

The NPS was used to assess knee pain based on a one-item questionnaire that assesses patients’ physical pain in the affected area using an eleven scoring levels ranging from 0 (no pain) to 10 (most severe pain) [34]. The LEFS consists of a 20 item measure of daily activities and function based on a five-point Likert scale [35], where difficulty levels for each item are classified into: extreme difficulty or inability to perform activity (0 point), quite a bit of difficulty (1 point), moderate difficulty (2 points), a little bit of difficulty (3 points), and no difficulty (4 points). The total LEFS score is the sum of points for the twenty items and ranges from 0 (i.e. minimum functional activity) to 80 points (i.e. maximum functional activity). The International Knee Documentation Committee scale (IKDC) is another subjective questionnaire consisting of 18 items that measure knee symptoms, function and sport activities [36]. The total IKDC score ranges from 0 to 100, with higher scores reflecting a better condition. The IKDC form includes three main sections: symptom, sport activities, and function. The three sections are combined in one total score. The subjective percentage improvement rating scale was based on the following question: *“Compared to your condition prior to the procedure, what percent difference have you seen in your condition?”* The patients’ response ranged from −100 % (denoting “worst condition”) to 100 % (indicating “best condition”). A score of zero would indicate no change.

### Statistical analysis

A receiver operating characteristics (ROC) curve analysis was used to determine the optimal threshold for dichotomizing cell counts into higher and lower cell count groups. Using a composite variable based on the NPS and LEFS (see Outcomes of Interest), a ROC curve analysis identified the level at which sensitivity and specificity for composite pain and functional improvement was maximized. To avoid having two different cut-points for the cell count, a composite variable of functional improvement and pain was chosen for the analysis rather than separately evaluating pain and functional improvement. Any improvement to the composite variable was defined as achieving the minimum important differences in both the NPS and LEFS, where a minimum important difference was indicated by a 9-point increase on the LEFS and a 2-point decrease on the NPS [34, 35]. A lack of improvement was defined as a failure to achieve any of these changes.

Mean and standard deviation of the baseline and follow-up scores are described, as well as the differences between these two scores. Intra-group changes from the baseline were analyzed using the signed rank test. An analysis of covariance (ANCOVA) was used to estimate the adjusted means, controlling for the associated baseline score and potential confounding factors. The same multivariate analysis was conducted on the four outcome (dependent) variables including: NPS, LEFS, IKDC and subjective improvement rating scale. ANCOVA models were tested for the homogeneity of variance, homogeneity of regression, and normal distribution of residuals. Homogeneity of variances was tested using Levene’s tests, and homogeneity of regression was tested by introducing interaction terms to the models and testing their significance. Models’ residuals were plotted using histograms and Q-Q plots, and scatter plots for residual and predicted values were also created. Plots were examined for normality, presence of outliers, or unusual patterns. Spearman correlation was also used to test the correlation between cell doses as a continuous variable with changes in clinical scales’ scores.

An analysis was also performed to assess the differences in baseline characteristics between patients who responded to the follow-up surveys and those who did not. This analysis included only procedures that had available data for each respective clinical scale. Wilcoxon rank sum, chi square, or Fisher exact tests were used when appropriate. Data were analyzed using SAS 9.4 software (SAS Institute Inc. 2014: Cary, NC). Statistical significance was considered at *p*-value ≤ 0.05. Post-hoc power analysis was performed using G*Power 3.1 software.

## Results

**Fig. 1:**
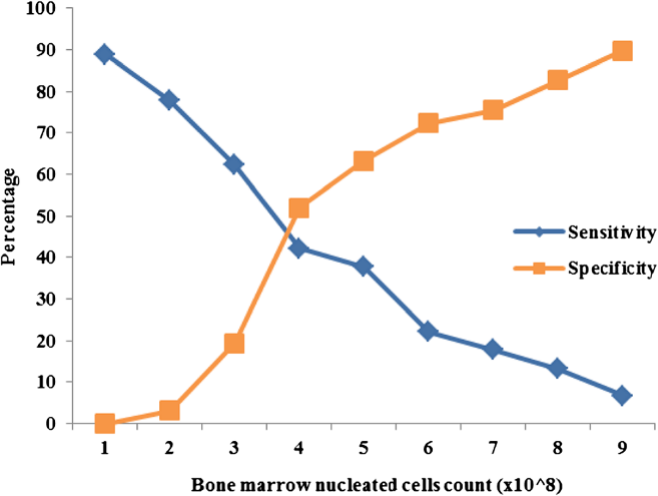
Sensitivity and specificity for composite pain and functional improvement as predicted by the nucleated cell count and examined by the receiver operating characteristics curve analysis

**Fig. 2:**
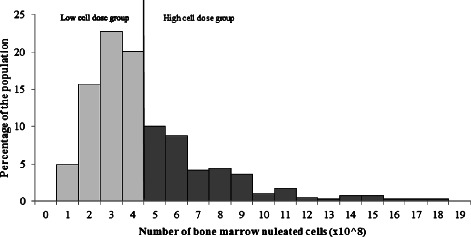
Nucleated cell count of the study population. Lower nucleated cell count group was categorized as ≤ 4 × 10^8^ cells; whereas in the higher cell count group, there were > 4 × 10^8^ nucleated cells

Between the periods of August 2010 and February 2014, a total of 373 patients received treatment in 424 knee joints. Cell count information was available for 409 of the procedures. Optimal sensitivity and specificity for pain and functional improvement was estimated via ROC at 4 × 10^8^ cells, and served as the value to discriminate between the higher and lower cell count groups (Fig. 1). There were 185 procedures (*n* = 170 patients) in the higher cell count group (>4 × 10^8^ cells), and 224 procedures (*n* = 188 patients) in the lower cell count group (≤4 × 10^8^ cells). Based on the radiological data, 55.6 and 59.4 % of the lower and higher cell count groups had early stage knee osteoarthritis (KL grade 1) (Table 1). The distribution of cell counts is presented in Fig. 2, and baseline characteristics of the higher and lower cell count groups are reported in Table 1.

**Table 1:** Baseline characteristics and the statistical significance of the differences between the lower and higher cell count groups (NC = nucleated cells, SD = standard deviation, KL = Kellgren-Lawrence)

	NC ≤ 4 × 10^8^ cells			NC > 4 × 10^8^ cells			*P*-value
	N	Mean	SD	N	Mean	SD	
Age	224	54.5	12.8	185	50.2	15.6	.003
BMI	207	25.4	3.8	168	25.9	3.9	.241
NC Count (X10^8^)	2.2	2.7	83.6	185	6.8	2.8	<.001
Gender	224			185			.001
Male	143	63.8 %		140	75.7 %		
Female	81	36.2 %		45	24.3 %		
Severity grade	160			128			.801
KL1	89	55.6 %		76	59.4 %		
KL2	48	30 %		36	28.1 %		
KL3-4	23	14.4 %		16	12.5 %

The total number of participants responding to the NPS, LEFS and IKDC questionnaires was 116, 94, and 47, respectively; these frequencies represent subjects who had available baseline and follow-up data for the respective clinical scale. There were no significant differences between the responders and non-responders to questionnaires with regard to demographics or baseline metrics. A reduction in pain following treatment was statistically significant in both treatment groups, as the NPS decreased by 1.5 and 0.9 in the higher and lower cell count groups, respectively (*P* < 0.001 and *P* = 0.006). There were positive changes in both the LEFS and IKDC scales in both groups as well; the LEFS scale improved by 8.9 and 4.8 (*P* = 0.002 and 0.001), and the IKDC improved by 14.2 and 19.7 (*P* < 0.001 and *P* = 0.004), for the higher and lower cell count groups, respectively.

After adjusting for potential confounding factors (see Tables 2, 3), the outcomes for the higher and lower cell count groups were compared. A difference in the NPS means between the two cell count groups was statistically significant (higher cell count group mean = 1.6, versus lower cell count group mean = 3.2; *P* < 0.001). In contrast, a comparison between the adjusted means for LEFS and IKDC scores indicated no significant differences between the higher and lower cell count groups. Spearman correlation test showed that only changes in NPS scores were correlated with cell dose as a continuous variable with a borderline statistical significance (correlation coefficient = −0.13, *P* = 0.081). Other clinical scales did not show significant correlation with cell dose.

**Table 2:** Baseline and follow-up means of the clinical scales, significance of intra-group changes and follow-up duration in months (NC = nucleated cells, SD = standard deviation, LEFS = lower extremity functional scale, NPS = numeric pain scale, IKDC = international knee documentation committee scale, P-values are for intra-group changes, improvement rating = percentage improvement rating scale)

	NC ≤ 4 × 10^8^ cells				NC > 4 × 10^8^ cells			
Variable	N	Mean	SD	*P*-value	N	Mean	SD	*P*-value
NPS								
Baseline	67	4	2		49	3.1	1.8	
Follow-up	67	3.1	2.5		49	1.7	2.1	
Change	67	−0.9	2.7	.006	49	−1.5	2.5	<.001
Follow-up duration	67	10.6	8.5		49	11	8.6	
LEFS								
Baseline	57	48.8	13.9		37	50.7	13.9	
Follow-up	57	53.5	18.7		37	59.6	17	
Change	57	4.8	16.7	.001	37	8.9	15.3	.002
Follow-up duration	57	8.5	6.5		37	8.5	6.1	
IKDC								
Baseline	25	45	16.1		22	55.9	16.6	
Follow-up	25	64.7	18		22	70.1	16.7	
Change	25	19.7	15.5	<.001	22	14.2	14.7	.004
Follow-up duration	25	6.3	7.5		22	5.7	3.5	
Improvement rating								
Follow-up	137	47	37.1		103	48.3	41.3	
Follow-up duration	137	14.8	11.6		103	13.4	10.7

**Table 3:** Means of the last follow-up scores of clinical scales adjusted for baseline score of the respective scale, follow-up time, age, BMI, gender, and severity grade (NC = nucleated cells, SE = standard error, LEFS = lower extremity functional scale, NPS = numeric pain scale, IKDC = international knee documentation committee scale, improvement rating = percentage improvement rating scale)

	NC ≤ 4 × 10^8^ cells			NC > 4 × 10^8^ cells			
	N	Mean	SE	N	Mean	SE	*P*-value
NPS	67	3.2	0.3	49	1.6	0.3	<.001
LEFS	57	53.4	2.4	37	58.7	2.7	.114
IKDC	25	68.3	3.7	22	68.4	3.9	.989
Improvement rating	137	46.4	3.7	103	50.9	4.3	.372

## Discussion

Consistent with the previously published results from our group and others, significant improvement of pain and function was observed in osteoarthritis knee joints following injection with BMC at both higher and lower doses of nucleated cells [8, 37]. In both groups, most knee joints were diagnosed with early-stage knee osteoarthritis. Although a higher cell count dose was associated with a greater reduction in pain, there were no significant differences in functional improvement including the LEFS and IKDC, and the subjective improvement rating scale following injection with either a higher or lower cell count.

Although absolute difference in pain outcomes between higher and lower doses was less than two points, the minimum important difference defined in the literature; the NPS score in the higher dose group was 50 % lower than that in the lower dose group, a difference which is considered clinically significant [38] especially given the fact that the comparison is made between two low means; therefore we do not expect to see a high absolute difference in scores despite the highly significant difference as a percentage.

Prior studies of cell dose and therapeutic efficacy of the BMC injectate have been inconclusive. In animal studies, intra-articular injection of either BMC (lower MSC content) or culture-expanded MSCs (higher MSC content) facilitated regeneration of damaged cartilage; however, neither approach showed any superiority in therapeutic efficacy [39]. Clinical studies in humans have demonstrated mixed results, with one study indicating the superiority of higher concentrations of MSCs at repair of cartilage and meniscus injury [40], and another study indicating greater efficacy at lower cell concentrations of culture-expanded allogeneic cells [41]. The findings from the latter study may be explained by a greater host immune response resulting from the higher-doses of allogeneic cells [26].

Previous work investigating MSC concentration in the reduction of discogenic low back pain, revealed similar findings with patients receiving higher cell concentration intra-discal injections reporting a statistically significant greater reduction in pain compared to lower cell concentrations [23]. The findings of the present study are unique; prior studies have described the use of BMC for treatment of symptomatic osteoarthritis, but there are none that have examined a cell dose response, or identified an optimal threshold count of nucleated cells for maximizing clinical outcome [31].

Conceptually, it makes sense that a higher BMNC count equates to better pain relief than a lower cell count. Nucleated cell count is a proxy for the total number of MSCs, and MSCs are the cells that contribute to regeneration of intra-articular cartilage [25, 42]. Unlike studies that have focused on culture expanded MSCs, the number of these cells in BMC is much smaller [24]. Hence, dose may be more critical in some patients with poor MSC counts in bone marrow, which is common in older patients [21]. Additionally, BMC constituents including hematopoietic stem cells, T-lymphocytes, B-lymphocytes, monocytes, macrophages, epithelial progenitor cells and platelets, are all capable of producing growth factors and cytokines that together may support a microenvironment that promotes proliferation and functional differentiation of MSCs as well as cartilage repair [43]. For example, co-culture of MSCs with Monocytes has been shown to increase chondrogenic differentiation capacity [12]. Finally, MSCs have been shown to reduce pain in animal models by the release of TGF-beta [44].

It is important to note that the observed treatment’s effect may be attributed to the platelet component of injections; PRP and PL have been shown in numerous studies to improve the symptoms associated with mild-moderate knee osteoarthritis [45–47]. However, the clinical efficacy of PRP therapy is transient, and relief from pain and function improvement declines to baseline between 6 and 24 months after treatment [48, 49]. Further, the efficacy of PRP therapy is limited in moderate and severe osteoarthritis, versus mild osteoarthritis [7, 50, 51]. Finally, the significant difference in pain score associated with the higher nucleated cell group in this study reveals that varying MSC dose, within a standardized protocol of platelet injections, has an impact on treatment outcome.

Limitations of the present study are typical of registry studies; there was no placebo control group, and there was no randomization of the patients into the lower and higher cell dose groups. As a result it is possible that uncontrolled for confounding factors or even a placebo effect may account for the results. It is unlikely that the positive results observed for both cell count groups were due to response bias; the analysis of the non-responders indicated no significant differences with regard to age, gender, weight, or baseline severity in comparison with the responders. Also the lack of significance of the differences in the functional outcome scales may be due to limited statistical power. Another limitation of this study is that the ROC analysis and ANCOVA models were performed on data subsets obtained from the same population. ROC analysis was performed on subjects who had available baseline and follow-up scores for both LEFS and NPS scales. ANCOVA models included subjects who had available data for all covariates, in addition to the baseline and follow-up scores of the respective outcome scale. Therefore, future research on different populations is needed to confirm our study’s findings. Sufficiently powered randomized placebo-controlled trials are needed to validate and expand on these preliminary results.

## Conclusion

Improved function and reduced pain was observed in patients treated with BMC injectate regardless of cellular dose; however, patients receiving a higher concentration of cells reported a better pain outcome in comparison with the lower dose group. These findings indicate that cell dose may be a factor governing clinical outcomes in autologous BMC treatment of knee osteoarthritis. Further studies using randomized and placebo-controlled design are needed.

## Abbreviation

ANCOVA: Analysis of covariance
BMC: Bone marrow concentrate
BMNC: Bone marrow derived nucleated cells
HSCs: Hematopoetic stem cells
IKDC: International knee documentation committee
KL: Kellgren-Lawrence
LEFS: Lower extremity function scale
MSC: Mesenchymal stem cell
MRI: Magnetic resonance imaging
NPS: Numeric pain scale
NSAID: Non-steroidal anti-inflammatory drug
PL: Platelet lysate
PRP: Platelet-rich plasma
ROC: Receiver operating characteristic
TKR: Total knee replacement
